# Relaxation dynamics of generalized scale-free polymer networks

**DOI:** 10.1038/s41598-018-21968-9

**Published:** 2018-02-27

**Authors:** Aurel Jurjiu, Deuticilam Gomes Maia Júnior, Mircea Galiceanu

**Affiliations:** 1 Department of Condensed Matter Physics and Advanced Technologies, Faculty of Physics, Babes-Bolyai University, Street Mihail Kogalniceanu 1, 400084 Cluj-Napoca, Romania; 2 Departamento de Física, Universidade Federal do Amazonas, 69077-000 Manaus, Brazil

## Abstract

We focus on treelike generalized scale-free polymer networks, whose geometries depend on a parameter, *γ*, that controls their connectivity and on two modularity parameters: the minimum allowed degree, *K*_*min*_, and the maximum allowed degree, *K*_*max*_. We monitor the influence of these parameters on the static and dynamic properties of the achieved generalized scale-free polymer networks. The relaxation dynamics is studied in the framework of generalized Gaussian structures model by employing the Rouse-type approach. The dynamical quantities on which we focus are the average monomer displacement under external forces and the mechanical relaxation moduli (storage and loss modulus), while for the static and structure properties of these networks we concentrate on the eigenvalue spectrum, diameter, and degree correlations. Depending on the values of network’s parameters we were able to switch between distinct hyperbranched structures: networks with more linearlike segments or with a predominant star or dendrimerlike topology. We have observed a stronger influence on *K*_*min*_ than on *K*_*max*_. In the intermediate time (frequency) domain, all physical quantities obey power-laws for polymer networks with *γ* = 2.5 and *K*_*min*_ = 2 and we prove additionally that for networks with *γ* ≥ 2.5 new regions with constant slope emerge by a proper choice of *K*_*min*_. Remarkably, we show that for certain values of the parameter set one may obtain self-similar networks.

## Introduction

Recent years have seen a growing interest in hyperbranched molecules, polymer structures without loops and hence, topologically speaking, tree-like^1–3^. It is well known the fact that the insertion of branch points into a polymer chain alters drastically its physical properties. The control of branching is an important issue in polymer synthesis and has led to the development of new molecules with complex architectures. Hyperbranched polymers can be obtained from different reaction pathways, therefore their architecture varies from completely ordered structures such as dendrimers, star polymers or regular fractals to disordered structures, such as irregular Cayley-trees or random hyperbranched fractals. Evidently, the synthesis of perfectly regular dendrimers is by far more demanding than that of usual hyperbranched macromolecules, for which in batch reactions one accepts a certain polydispersity and also a high pattern diversity. In this paper, we introduce a new treelike structure that is able to map the transition from a predominant starlike architecture to a linear or dendrimerlike topology. This transition is realized for a treelike scale-free polymer network by alternating two modularity parameters, namely the minimum and the maximum allowed degree. This network will be called as *generalized scale-free polymer network* (GSFPNs). In order to explain the peculiar properties of real networks, such as World Wide Web^4,5^, the author collaboration network of scientific papers^6^, and metabolic networks in biological organisms^7^ to name only a few, many theoretical models were developed. All these models of scale-free networks^8–12^ led to a power-law degree distribution for high degrees. In this work we generalize the known models of scale-free networks by considering the range of allowed degrees as flexible and focus on a basic problem, namely on how the static and dynamic features of a polymeric material are related to its geometry. Our aim is to show how the fundamental feature of polymers, the connectivity, affects its static and dynamic properties. If the minimum and the maximum permitted degrees are varied a greater amount of possible architectures are encountered, ranging from starlike to dendrimerlike or linear topology. In this way the control of the parameters, which is nothing else than the control of branch points and of the maximum permitted connections of a monomer, allows us to predict the type of the obtained structure; it is a long linear chain with small side chains attached or it is irregular (modified) dendrimer. To a great extent, the latter may be viewed as possible cheaper alternatives to the more precise dendrimers.

We perform our calculations in the framework of the generalized Gaussian structures (GGSs) model which represents the extension of the Rouse model^13^, developed for linear polymer chains, to polymer systems of arbitrary topologies and which highlights the most fundamental feature that distinguishes macromolecules from simple liquids, namely the polymer’s connectivity. This leads to a dynamical theory in which excluded volume constrains, hydrodynamic interactions, and entanglements effects are neglected. We note that in rather dense media, such as dry polymer networks and polymer melts^13–16^, the excluded volume effects are often screened. In turn, the entanglement effects are not significant in the case of polymer networks with high densities of cross-links, meaning that the network strands between the cross-link points are rather short, which is also the case of our generalized scale-free polymer networks. It is important to mention that there are models taking into account some of the above-cited interactions. The hydrodynamic interactions, which are solvent-mediated interactions, can be included explicitly in a preaveraged Oseen manner: the so-called Zimm model^17–19^. The excluded volume interactions and entanglements effects are considered in theoretical polymer models^20,21^ and the stiffnes effect are included in semiflexible models^22–25^. In spite of the fact that it disregards several important features, the Rouse model captures the dynamic properties of many systems, including concentrated polymer solutions and melts of rather short chains.

The advantage of using the GGS model is that it allows one to explore very efficiently the structural properties, as well as the static and dynamical properties of arbitrarily connected polymers by making use of the eigenvalues and eigenvectors of the connectivity matrix. The influence of the connectivity on the dynamics of the GSFPNs is studied by investigating the behavior of the relaxation quantities: the averaged monomer displacement under locally acting forces and the mechanical relaxation moduli (storage modulus and loss modulus)^14,16,26^. Additionally, the static properties of the GSFPNs will be studied by analyzing the behavior of the diameter and degree correlations. In this aspect, fundamental in the study of relaxation patterns is the intermediate time/frequency region of the relaxation quantities, where the topological details of the structure are revealed. Due to the fact that the intermediate region is bounded by large crossover domains and in order to unveil the influence of the topology on the dynamic quantities it is necessary to consider relatively large structures. Consequently, this leads to large connectivity matrices whose exact numerical diagonalizations get costly. Furthermore, in order to ensure an accurate statistics the quantities to be presented are ensemble averaged; for each desired network size we have generated and diagonalized hundreds networks of that size, leading to a huge computational time. In general, the connectivity matrix, being the discrete version of the Laplacian operator, is greatly used in many areas of science; for instance, in graph theory applied to biological systems^27^, reaction-diffusion systems^28^, in the study of fluorescence depolarization under dipolar quasiresonant energy transfer^29,30^, the dielectric relaxation functions^31^, and the NMR relaxation functions^32,33^.

Due to the continuous advancement in polymer synthesis and analysis, new macromolecules or supramolecules with very complex architectures and tunable properties have been synthesized. Paralleling the advancement on polymers synthesis, the Rouse-type approach has been successfully applied in the theoretical works devoted to the study of polymers with more complex architecture, such as dendrimers and their derivatives^26,34–37^, star polymers^34^, hyperbranched or fractal polymer networks^31,38–40^, multilayered or multihierarchical polymer networks^41–43^, small-world polymer networks^44–46^ and scale-free polymer networks^8^. The present work extents all these studies by considering the treelike generalized scale-free polymer networks and we systematically monitor the influence of the modularity parameters on the topology and dynamics of these complex networks. Networks similar to those proposed by us have been already experimentally synthesized. Among them prominent are POSS polymers^47–49^, complex supramolecular dendritic polymer networks in melt state^50,51^, diblock copolymer micelles^52^, and the multiarm star-shaped polymer and their corresponding self-assemblies^53–55^.

The static and dynamic properties of many real networks^56^, such as the internet, citation networks, transportation networks, social networks, neural networks or ecological networks can be understood by implementing a scale-free network model. A complete scientific study of real networks combines concepts from mathematics, physics, chemistry, biology, computer science, economy or social sciences. Therefore, the study of a generalized scale-free network model is of great importance and leads to interdisciplinary scientific advances that generate new avenues of research related, in particular, to chemical physics but, also, to different areas of science.

The rest of the paper is structured as follows: In Methods we describe our algorithm that creates the networks from a scale-free degree distribution with two additional modularity parameters and we briefly remind the formalism of GGS and solve the system of Langevin differential equations that governs the relaxation dynamics. In Results we compute several network related structural quantities, such as the degree distribution, the diameter, and the degree correlations. Here, we also present important aspects regarding the eigenvalue spectrum of the connectivity matrix, having as focus the influence of the three parameters: *γ*, *K*_*min*_, and *K*_*max*_. We monitor the relaxation patterns of the average monomer displacement and the two mechanical relaxation moduli. The Discussion will end this paper.

## Methods

In this section, we first present our model of treelike scale-free polymer network with two modularity parameters and then we provide a brief introduction to our theoretical framework of polymer relaxation dynamics.

### Construction model

In the theory of complex networks, the degree *k* of a node is defined as the number of links that connect this node with its nearest neighbors. A general characteristic for the models of scale-free networks is that the degree distribution obeys a power-law:

1 $${\bar{p}}_{k}\propto {k}^{-\gamma },$$

where ${\bar{p}}_{k}$ is the probability of having a node with degree *k* and *γ* measures the density of network’s connections. Networks with the degree distribution (1) can be obtained by employing some construction mechanisms^9,11^ or by first assuming that the nodes must obey the distribution (1) and then start the construction algorithm^57^. In this article we generalize the last method by the introduction of two modularity parameters. This is performed by assuming the probability that the degree of a node equals *k* has the following expression:

2 $${p}_{k}=\{\begin{array}{cc}\frac{{k}^{-\gamma }}{{\sum }_{j={K}_{min}}^{{K}_{max}}{j}^{-\gamma }}, & {K}_{min}\le k\le {K}_{max}\\ 0, & k < {K}_{min},\end{array}$$

where *K*_*min*_ represents the minimum allowed degree and *K*_*max*_ is the maximum allowed degree. These two modularity parameters, together with *γ*, allow a more general study of possible network topologies, which can be obtained by implementing Eq. (2). As a consequence, the model developed in^8,57,58^ becomes only a particular case of our model, more exactly it corresponds to (*K*_*min*_, *K*_*max*_) = (2, *N* − 1), where *N* is the size of the network. The sum in the denominator of Eq. (2) keeps the total probability equal to 1. It is worth to stress that in our model the parameter *γ* can have any positive nonzero value. The construction procedure starts by fixing the values of *γ*, *K*_*min*_, and *K*_*max*_ and the probabilities *p*_*k*_ are calculated, according to Eq. (2). In the end the algorithm builds a treelike network, whose nodes follow the degree distribution (2), except the peripheral nodes.

**Figure 1:**
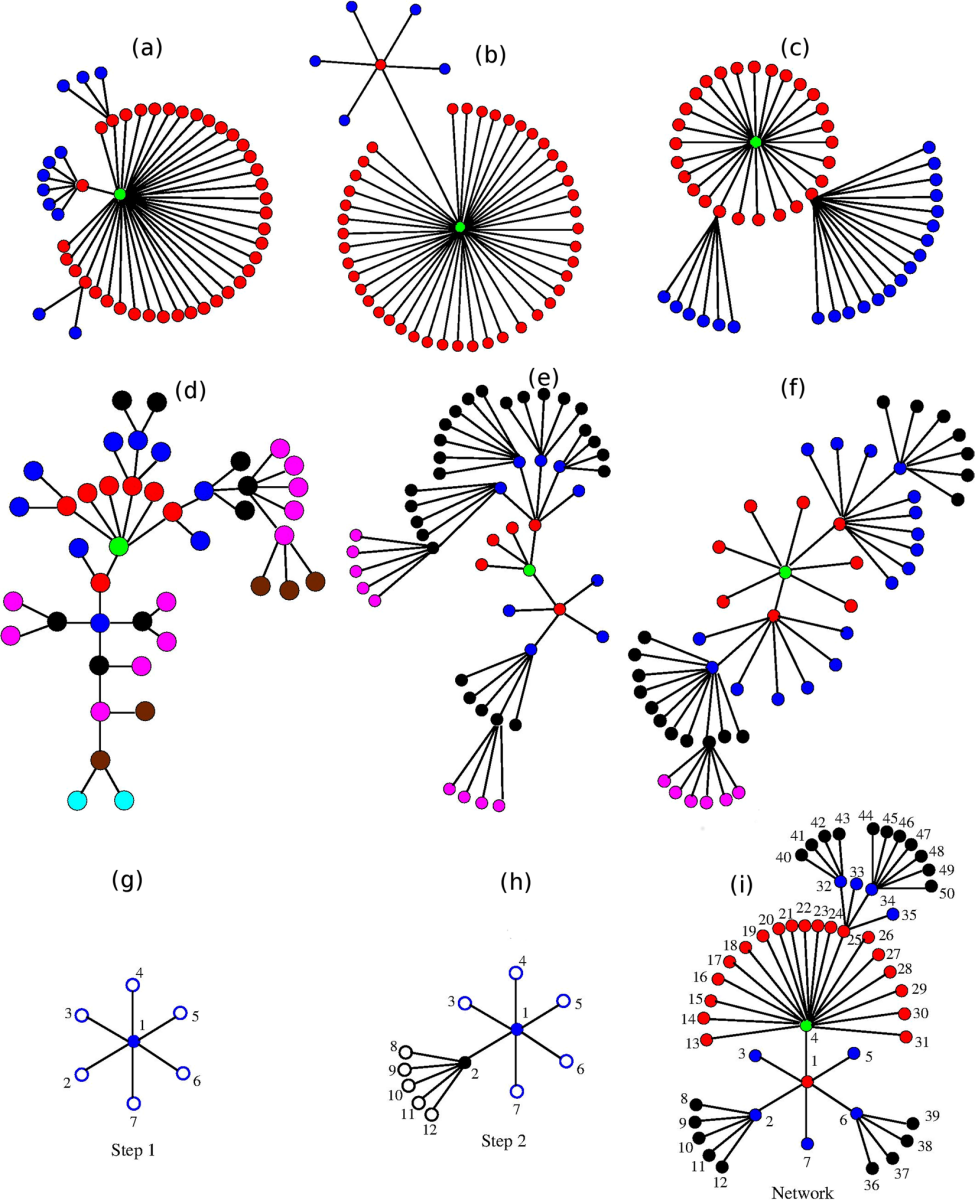
Realizations of generalized scale-free networks with the parameter set (*γ*, *K*_*min*_):(1, 2), (1, 4),(1, 6) (**a**–**c**) in the figure), and (4, 2), (4, 4), (4, 6) (**d**–**f**). Construction procedure in detail for the parameters (*γ*, *K*_*min*_) = (2.5, 4) (**g**–**i**).

Figure 1(a–f) displays several particular realizations of our algorithm for GSFNs with *N* = 50 nodes and fixed *K*_*max*_ = *N* − 1. The parameter *γ* equals 1.0 in the first row and 4.0 in the second row. In order to highlight the effect of the parameter *K*_*min*_ on the topology of the network we choose, in each row of the figure (for instance, panels (a–c)), the parameter *K*_*min*_ = 2, 4, and 6, from left to right. From these examples one can notice that by increasing *γ* for GSFNs with *K*_*min*_ = 2, compare for instance Fig. 1(d) (*γ* = 4.0) with Fig. 1(a) (*γ* = 1.0), we obtain networks with higher longest linear path and with a smaller amount of nodes with very high degree. On the other hand, by increasing the value of *K*_*min*_ and keep *γ* constant, compare for instance Fig. 1(d) (*K*_*min*_ = 2) with Fig. 1(f) (*K*_*min*_ = 6), the longest linear path and the emergence of nodes with high degree is more probable. This is a direct consequence of increasing the minimum allowed degree, *K*_*min*_. It is evident from the first two rows of Fig. 1 that for GSFNs with high *γ* one obtains networks with more nodes with high degree by simply increasing the value of *K*_*min*_. Their topologies are similar with GSFNs that have smaller values for both parameters *γ* and *K*_*min*_. Not shown in the Fig. 1 but also the parameter *K*_*max*_ is varried throughout the article. It is worth to stress that by switching on the parameter *K*_*max*_ such that *K*_*min*_ = *K*_*max*_ one gets networks formed by nodes with the same degree, similar to modified dendrimers.

Let us exemplify the construction algorithm for a particular network with *γ* = 2.5 and *K*_*min*_ = 4, last row of Fig. 1. In these subfigures the numbering is according to the chronological order in which the nodes are added. The growth begins with vertex 1, to which one chooses randomly its number of connections, i.e. its degree, from the degree distribution (2). For this particular realization the degree of node 1 is equal to 6. Thus, we add six new vertices and all of them have a direct connection to node 1. In the second construction step, displayed in panel 1 (h), we choose randomly one of the open nodes and we give its degree accordingly to the degree distribution (2). In our case the chosen node is 2 and its degree is 6. At this point we have to add only five new nodes, numbered from 8 to 12 in the subfigure, because the node 2 already has a direct connection with node 1. This procedure is iterated until the desired number of nodes, *N*, is reached. When the desired size of the network is achieved the growth is stopped and we assign to all remaining open vertices the degree one. In our example we stopped the construction when we reached the preset value *N* = 50, resulting the network displayed in Fig. 1(i). By using this algorithm the construction never stops by itself due to the lack of open vertices and every internal node has at least *K*_*min*_ and a maximum of *K*_*max*_ neighbors, while all the peripheral nodes are open, having degree 1. We apply our algorithm that construct treelike GSFNs to the relaxation dynamics of polymer networks. However, we stress that it can be applied with great success to other real complex networks, such as ecological networks^59,60^, epidermic spreading^61^ or transport networks^56^, to name only a few. It would be extremely interesting to monitor the influence of our two additional parameters *K*_*min*_ and *K*_*max*_ on network’s robustness or on diffusive processes.

### Theoretical model

Gaussian models are very valuable because they allow one to study static and dynamic quantities in the framework of linear algebra. The method of choice in this paper is that of the generalized Gaussian structures (GGS)^26,34,62^ which, as we already mentioned in Introduction, successfully extended the classical Rouse model^13^ to systems of arbitrary topology. Given that the procedure of GGS was explained in detail in refs^26,34,62^, we mainly summarize the basic concept and the main formulas concerning the relaxation dynamics patterns. The GGS consist of *N* identical beads, which are joined together by elastic springs with the same elasticity constant *K*. These elastic (entropic) springs obey a Gaussian statistics. In this model the solvent or the surrounding medium is replaced by a continuum, which is felt by all the beads through the viscous friction and the stochastic (or random) forces. Here we consider a homogeneous situation, in which all the beads experience the same friction constant *ζ* with respect to the surrounding viscous medium. Any polymer network’s configuration is given by a set of position vectors {R_*n*_}, where R_*n*_(*t*) = (*X*_*n*_(*t*), *Y*_*n*_(*t*), *Z*_*n*_(*t*)) is the three-dimensional position vector of the *n*th bead measured at time *t*. The dynamics of the whole network is described by the set of *N* linearly independent Langevin equations, which for a bead *i* has the form:^14,34,63^

3 $$\zeta \frac{\partial {{\bf{R}}}_{i}(t)}{\partial t}+K\sum _{j=1}^{N}{\bf{A}}{{\bf{R}}}_{j}(t)={{\bf{f}}}_{i}(t)+{{\bf{F}}}_{i}(t),$$

where *ζ* = 6*πρa* is the friction constant of the beads (usually formulated in terms of an effective radius *a* and viscosity of the solvent *ρ*), *K* = 3*k*_*B*_*T*/*l*^2^ is their elasticity constant (with *T* being the temperature, *k*_*B*_ the Boltzmann constant, and *l*^2^ the mean square end-to-end distance for an unstretched bond) and **A** is connectivity matrix. In Eq. (3), **f**_*i*_ represents the stochastic force that acts on the *i*th bead and due to the fluctuation-dissipation theorem this force is connected with the dissipative force (or friction). Any external force acting on the bead *i* is represented by **F**_*i*_. The solution of Eq. (3) can be write as

4 $${{\bf{R}}}_{i}(t)=\frac{1}{\zeta }{\int }_{-\infty }^{t}dt^{\prime} \exp [-\frac{K}{\zeta }(t-t^{\prime} ){\bf{A}}][{{\bf{f}}}_{i}(t^{\prime} )+{{\bf{F}}}_{i}(t^{\prime} )]$$

and it can be verified by differentiating its right-hand-side with respect to *t*. The connectivity matrix **A** = **A**_*ij*_, also called as the Laplacian matrix^14,62^, stores the information about the topology of the GGS. This *N* × *N* matrix is symmetric and it contains the following elements: the nondiagonal elements *A*_*ij*_ equal −1 if the *i*th and *j*th beads are directly connected and 0 otherwise and the diagonal elements *A*_*ii*_ equal the number of connections of the *i*th bead. A direct consequence of these definitions is that *det***A** = 0, implying that (at least) one eigenvalue equals 0. The connectivity matrix A can be diagonalized **AQ** = **ΛQ**, where **Q** is the complete set of eigenvectors of **A** and **Λ** is the diagonal matrix whose elements are the eigenvalues *λ*_*i*_ of **A**. Knowing that any function of **A** can be written as^34^ *g*(**A**) = **Q** ⋅ *g*(**Λ**) ⋅ **Q**^−1^ we can write the displacement of the beads as

5 $${{\bf{R}}}_{i}(t)=\frac{1}{\zeta }{\int }_{-\infty }^{t}dt^{\prime} {\bf{Q}}\exp [-\frac{K}{\zeta }(t-t^{\prime} ){\boldsymbol{\Lambda }}]{{\bf{Q}}}^{-1}[{{\bf{f}}}_{i}(t^{\prime} )+{{\bf{F}}}_{i}(t^{\prime} )]\mathrm{.}$$

The expression of the displacement can be simplified by averaging over the random forces **f**_*i*_. These random forces arise due to the incessant collisions of the solvent molecules with the bead and are considered Gaussian processes with zero mean value 〈**f**_*i*_(*t*)〉 = 0 and 〈*f*_*iα*_(*t*)*f*_*jβ*_(*t*′)〉 = 2*k*_*B*_*Tζδ*_*ij*_*δ*_*αβ*_*δ*(*t* − *t*′) (with *α* and *β* denoting the *x*, *y*, and *z* directions and *i*, *j* correspond to monomers’ number). Furthermore, the GGS problem is linear and the different components (*X*_*i*_, *Y*_*i*_, *Z*_*i*_) decouple. We consider the motion of the GGS under a constant external force **F** = *F* ⋅ Θ(*t*) ⋅ **e**_*y*_, with Θ(*t*) being the Heaviside step function, which is switched on at *t* = 0 and acts on a single bead along the *y* direction. Thus, the mean displacement of the *i*-th bead in *y*- direction can be written as

6 $$\langle {Y}_{i}(t)\rangle =\frac{F}{\zeta }\sum _{j=1}^{N}{\int }_{0}^{t}dt^{\prime} {Q}_{ij}\exp [-\frac{K}{\zeta }{\lambda }_{j}(t-t^{\prime} )]{Q}_{ij}^{-1}\mathrm{.}$$

Now we average over all monomer positions and we obtain

7 $$\begin{array}{ll}\langle \langle Y(t)\rangle \rangle  & =\frac{1}{N}\sum _{i=1}^{N}\langle {Y}_{i}(t)\rangle \\  & =\frac{F}{N\zeta }{\int }_{0}^{t}dt^{\prime} {\bf{Tr}}({\bf{Q}}\exp [-\frac{K}{\zeta }(t-t^{\prime} ){\boldsymbol{\Lambda }}]{{\bf{Q}}}^{-1}),\end{array}$$

where **Tr** denotes the trace of the matrix. Using that the trace is invariant under cyclic permutations we obtain

8 $$\begin{array}{ll}\langle \langle Y(t)\rangle \rangle  & =\frac{F}{N\zeta }{\int }_{0}^{t}dt^{\prime} {\bf{Tr}}({\rm{\exp }}[-\frac{K}{\zeta }(t-t^{\prime} ){\boldsymbol{\Lambda }}])\\  & =\frac{F}{N\zeta }{\int }_{0}^{t}dt^{\prime} \sum _{n\mathrm{=1}}^{N}{\rm{\exp }}[-\sigma {\lambda }_{n}(t-t^{\prime} )],\end{array}$$

where $\sigma =\frac{K}{\zeta }$ is the bond rate constant. The previous equation can be integrated and the average monomer displacement takes the following form

9 $$\langle \langle Y(t)\rangle \rangle =\frac{Ft}{N\zeta }+\frac{F}{\sigma N\zeta }\sum _{n=2}^{N}\frac{1-{\rm{\exp }}(-\sigma {\lambda }_{n}t)}{{\lambda }_{n}}\mathrm{.}$$

The computational effort is reduced due to the fact that in the Rouse model the average monomer displacement depends only on the eigenvalues *λ*_*n*_ of the connectivity matrix **A**, but not on its eigenvectors. The behavior of 〈〈*Y*(*t*)〉〉 for extremely short times and for very long times is obvious. In the limit of very short times and for large *N* one has 〈〈*Y*(*t*)〉〉 = *Ft*/*ζ*, and for very long times one gets 〈〈*Y*(*t*)〉〉 = *Ft*/*Nζ*. From the physical point of view the interpretation is that for very short times only one bead is moving, whereas for very long times the whole network drifts, which increase the total friction from *ζ* to *Nζ*. In the intermediate time region the network topology of the GGS will play an important role, the behavior of 〈〈*Y*〉〉 depends on all the eigenvalues of the matrix **A**, except the eigenvalue, *λ* = 0. Because we are mainly interested in the slope of 〈〈*Y*〉〉 we set *F*/*ζ* = 1 and *σ* = 1.

Apart from 〈〈*Y*(*t*)〉〉, a quantity which may be accessed through micromechanical manipulations, classical mechanical experiments focus on the mechanical relaxation. Most rheological experiments probe the complex dynamic modulus G^*^(*ω*) or, equivalently, its real *G*′(*ω*) and imaginary *G*′′(*ω*) components known as the storage and the loss modulus^14,16^. For very dilute solutions and for *ω* > 0, the storage and loss modulus are given by (see also Eqs. (4.159) and (4.160) of Ref.^14^)

10 $$G^{\prime} (\omega )=\nu {k}_{B}T\frac{1}{N}\sum _{i=2}^{N}\frac{{\omega }^{2}}{{\omega }^{2}+{\mathrm{(2}\sigma {\lambda }_{i})}^{2}}$$

and

11 $$G^{\prime \prime} (\omega )=\nu {k}_{B}T\frac{1}{N}\sum _{i=2}^{N}\frac{2\sigma \omega {\lambda }_{i}}{{\omega }^{2}+{\mathrm{(2}\sigma {\lambda }_{i})}^{2}},$$

where *ν* represents the number of polymer segments (beads) per unit volume and *λ*_*i*_ are the eigenvalues of the connectivity matrix **A**. In these equations the sum runs over all the eigenvalues, except the vanishing eigenvalue (*λ*_1_ = 0), which corresponds to the whole translation of the system. Also, for concentrate solutions (when the entanglement effects are negligible) the Eqs (10) and (11) are still valid, the only change being in the value of the *νk*_*B*_*T*^16^. The factor 2 in the relaxation times *τ*_*i*_ = 1/2*σλ*_*i*_ arises from the second moment of the displacements involved in computing the stress^14^. For these moduli we are mostly interested in the slopes, thus we will compute the results in terms of the reduced storage and loss moduli by setting *νk*_*B*_*T*/*N* = 1 and *σ* = 1 in (10) and (11).

It is noteworthy to mention that in the GGS theory the considered rheological properties correspond to other experimental (non-mechanical) techniques. Besides mechanical viscoelastic experiments, one can also perform dielectric relaxation measurements, which constitute another well-established technique in polymer physics. In turn, the average monomer displacement under a constant external force is related to the mean-square displacement of a monomer on which no such force is applied.

## Results

In this section we focus on some structural properties of generalized scale-free networks. Then, we study the relaxation dynamics by considering physical quantities, such as the complex dynamic modulus and the average monomer displacement. Here, we monitor the influence of the modularity parameters *K*_*min*_ and *K*_*max*_ on the aforementioned quantities and on the eigenvalue spectrum.

### Network properties

#### Degree distribution

**Figure 2:**
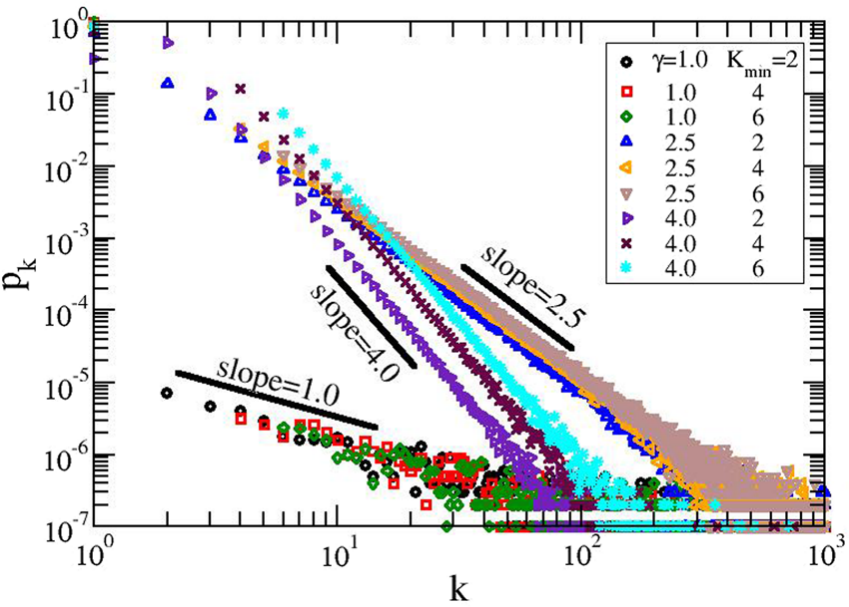
Degree distribution for GSFNs with *N* = 100000 and *S* = 100 realizations of the algorithm.

The degree distribution *p*_*k*_ of our constructed networks is shown in Fig. 2, facilitating a direct comparison with the theoretical prediction, Eq. (2). Displayed are the results obtained for the networks consisting of *N* = 100000 nodes and the number of realizations of the construction algorithm, *S* = 100. We kept constant the parameter *K*_*max*_ = *N* − 1 and vary the other modularity parameter *K*_*min*_ to 2, 4, and 6. For the parameter, *γ*, we have chosen three values, namely 1.0, 2.5, and 4.0. For very large values of the degree we obtain the usual fat tail behavior^9^, while for intermediate values we recover, from the slope, the theoretical predicted values of *γ*, for all *K*_*min*_-values. As expected, we get *p*_*k*_ = 0.0 for *k* < *K*_*min*_, except *p*_1_, i.e. nodes with degree 1, which corresponds to the peripheral nodes of our treelike networks.

#### Diameter

**Figure 3:**
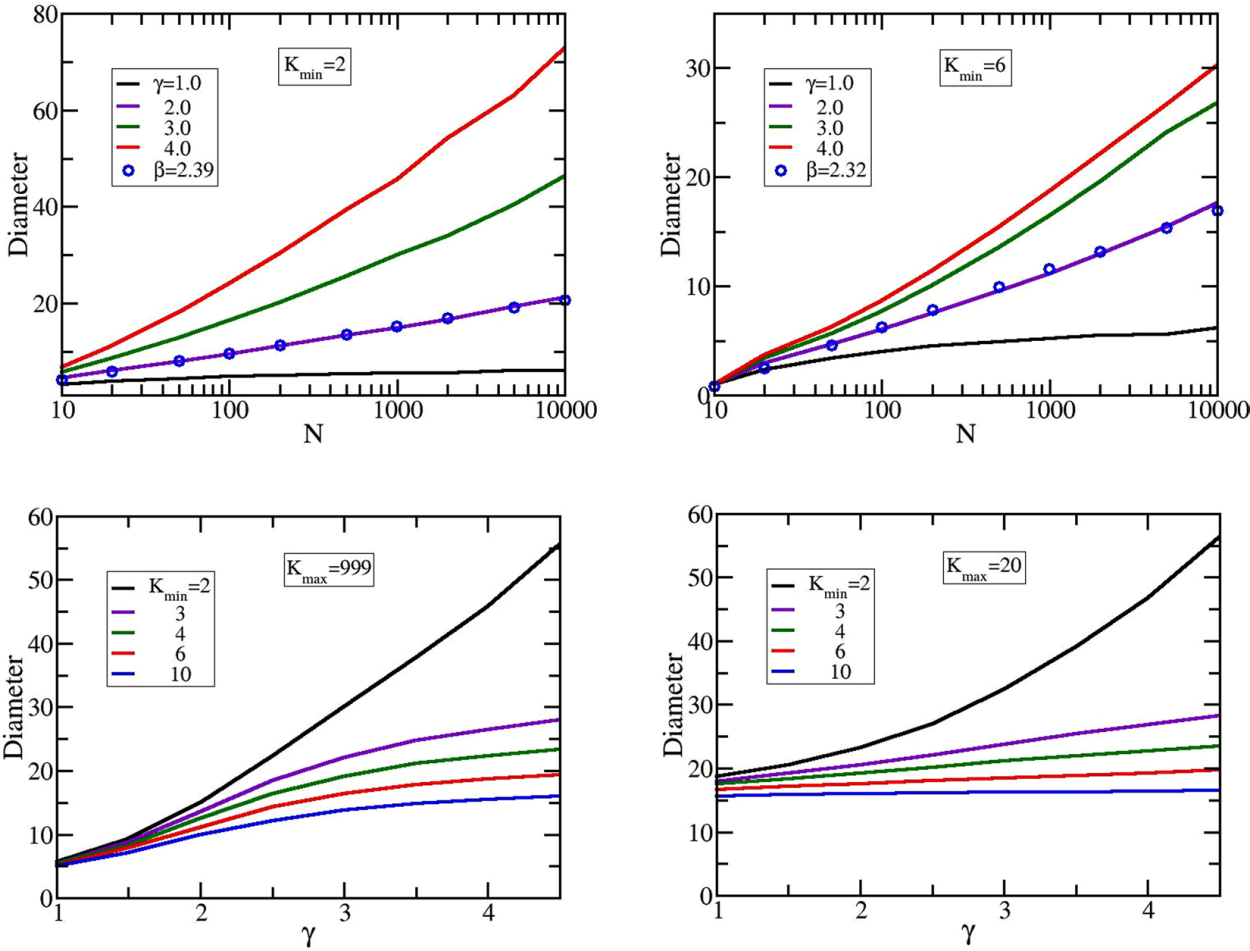
Diameter of GSFNs as a function of *N* (row above) and as a function of *γ* (row below).

The diameter of a network is defined as the maximum of the shortest distances between all possible pairs of nodes. In Fig. 3 we plot the diameter of different GSFNs. The upper panels of Fig. 3 display the diameter as a function of the network size, *N*, keeping constant the product *N* ⋅ *S* to 1000000, where *S* corresponds to the number of realizations of the algorithm. In the left panel we fixed *K*_*min*_ to 2 and in the right panel the lowest allowed degree is *K*_*min*_ = 6, while for both panels we have *K*_*max*_ = *N* − 1. In both upper panels, for *γ* = 2, one observes a logarithmic behavior of the diameter. This behavior reflects the increase of the dendritic-like segments. For a perfect dendrimer of functionality *f* and generation *G* the total number of nodes is $N=\frac{f\cdot {(f-\mathrm{1)}}^{G}-2}{f-2}$. After some algebraic calculations and considering a relatively large number of nodes one finds for the diameter of the dendrimer the following analytical expression, $diameter=2\cdot G\approx \frac{2}{lo{g}_{10}(f-\mathrm{1)}}lo{g}_{10}N\sim \beta lo{g}_{10}N$. From the logarithmic fitting of our data achieved for GSFNs with *γ* = 2.0, results *β* = 2.39 for *K*_*min*_ = 2 and *β* = 2.32 for *K*_*min*_ = 6, values that are very close to *β*_*D*_ = 2.36 of a dendrimer of functionality *f* = 8. However, it is worth to stress that similar logarithmical behavior was also determined for other small-world networks^46,56^. For other values of *γ* the logarithmic dependence vanishes and will be replaced by crossover behaviors towards two limiting cases. For very small *γ*s all the degrees are almost equally probable and thus one might obtain as the limiting structure a star with *N* − 1 arms, having its diameter non-dependent on *N*. For very high *γ*s the lowest degree is the most probable and thus we get as limiting structure a linear chain (for *K*_*min*_ = 2) or a sequence of coupled stars with the minimum functionality *K*_*min*_, both showing a linear dependence on *N*.

In the lower panels of Fig. 3 we display the diameter as a function of the networks’ connectivity parameter *γ*. In these panels, we kept constant the size of the network, *N* = 1000, and we fixed the parameter *K*_*max*_ to *N* − 1 (left panel) and *K*_*max*_ = 20 (right panel). Immediately apparent is an increase of the diameter’s size by increasing *γ*, supporting the transition behavior from a star-shaped network to a linearlike (or coupled stars in a linear manner for *K*_*min*_ ≠ 2) structure. For GSFNs with *K*_*max*_ = 999, displayed on left panel, one can notice that for *K*_*min*_ = 2 the diameter shows a power-law behavior with exponent 1.55 and for *K*_*min*_ > 2 we obtain a third order polynomial behavior. By decreasing the value of the modularity parameter *K*_*max*_ = 20 we observe an exponential behavior for *K*_*min*_ = 2 and for *K*_*min*_ > 2 the polynomial behavior maintains, but with an increase in the dominance of the linear term, easily observable for *K*_*min*_ = 10. As observed also from these subfigures, diminishing the value of *K*_*max*_ the diameter of GSFNs will change only for the values of *γ* smaller than 2.5. When the maximum allowed degree, *K*_*max*_, decreases the diameter increases due to the fact that nodes with very high degree (higher than *K*_*max*_) are forbidden. Not shown here, but it is important to mention that similar behaviors were observed also for intermediate values of *K*_*max*_.

#### Degree correlations

The degree correlations provide a better insight in the topology of the networks and it can be computed by the Pearson coefficient^46,56^. This quantity has values only in the interval [−1, 1], being equal to 0 if the network is uncorrelated. The negative values correspond to disassortative networks, while the positive values denote that the networks are assortative. A network is called assortative when the nodes with high degree are connected on average with nodes with high degree and nodes with low degree tend to stick together. The network is disassortative when the direct links occur on average between a node with high degree and a node with low degree, giving birth to a more star-like network or linearly connected star-like objects (having arms consisting of one or few number of nodes). The Pearson coefficient, denoted by *C*, depends on the networks’ parameters *γ*, *K*_*min*_, and *K*_*max*_ and is defined as^56^

12 $$C(\gamma ,{K}_{min},{K}_{max})=\frac{{S}_{1}\cdot {S}_{e}-{S}_{2}^{2}}{{S}_{1}\cdot {S}_{3}-{S}_{2}^{2}},$$

where *S*_1_ = ∑_*i*_*k*_*i*_, ${S}_{2}={\sum }_{i}{k}_{i}^{2}$, and ${S}_{3}={\sum }_{i}{k}_{i}^{3}$, with the summation being done over all the nodes. The sum *S*_*e*_ = 2 ⋅ ∑_(*i*,*j*)_*k*_*i*_*k*_*j*_ runs over all possible pairs of vertices *i* and *j* connected by an edge. In the last equations, *k*_*i*_ and *k*_*j*_ stand for the degrees of nodes *i* and *j*, respectively.

**Figure 4:**
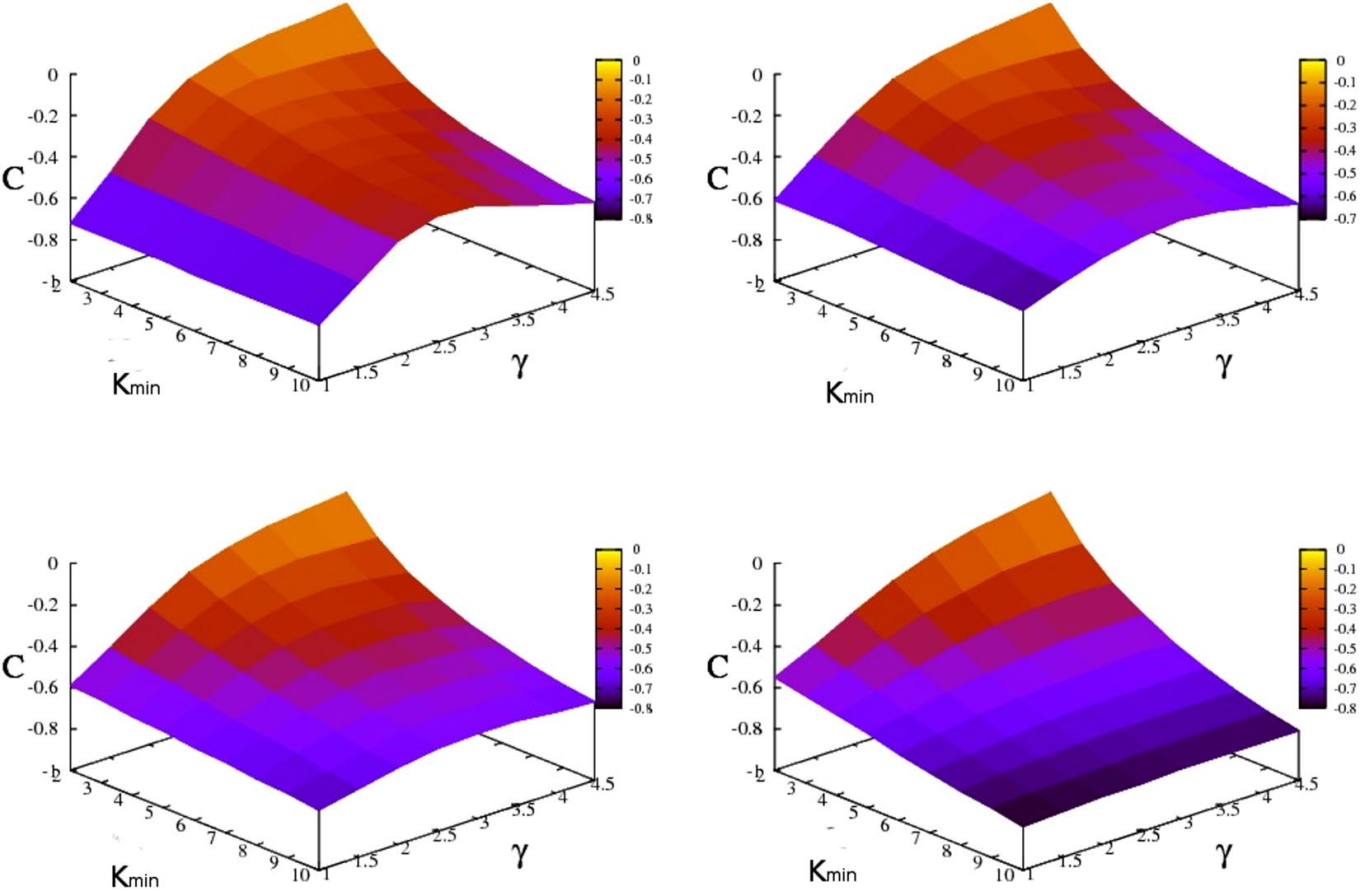
Pearson correlation coefficient *C*(*γ*, *K*_*min*_, *K*_*max*_) for GSFNs with *N* = 1000 and *K*_*max*_ equals *N* − 1 (top left), 100 (top right), 50 (down left), and 20 (down right).

Figure 4 presents the Pearson correlation coefficient *C* as a function of *γ* and *K*_*min*_ for generalized scale-free networks with *N* = 1000 nodes, while the other parameter *K*_*max*_ has been fixed to the values 999,100,50, and 20, respectively. Immediately apparent is that for our parameters’ choice our networks are disassortative for all parameter sets (*γ*, *K*_*min*_, *K*_*max*_). The lowest *C*-values are encountered for *γ* = 1.0, which correspond to networks that contain predominately nodes with very high degree connected mainly with nodes of low degree. The limiting case is a star, the central node being connected with *N* − 1 peripheral nodes, for which the Pearson coefficient is *C* =  − 1. By increasing the value of *γ* one obtains larger values of the Pearson coefficient, meaning that the percentage of connections between nodes with higher degree starts to increase, but still the dominant connections are those formed between nodes with higher degree and nodes with lower degree. For all the values of *K*_*max*_ the largest Pearson coefficient has been obtained for *K*_*min*_ = 2 and *γ* = 4.5, being equal to  − 0.09. Keeping constant *K*_*min*_ (for *K*_*min*_ > 5) and varying *γ* one observes the appearance of a local maximum in the region of *γ* ≈ 2.5, which is more pronounced for *K*_*max*_ = *N* − 1. This finding is perfectly justified by the fact that in the limiting case $\gamma \gg 1$ the algorithm creates GSFNs with nodes that are connected to many peripheral nodes, thus for very high *γ* the value of *C* becomes lower. At this point, it is crucial to remind that our networks are trees. Thus, there will be many open nodes, namely nodes with degree one, for which their degree was not chosen from the degree distribution (2) before reaching the system size *N*. Another important aspect is that the networks have finite sizes and in order to draw a clear statement regarding the assortativity one has to consider infinite sizes. However, with all these restrictions our networks show assortative mixing by degree similar to other real complex networks, except the social networks, see Table 8.1 of ref.^56^. For the particular case of very large *γ* we are able to calculate analytically the Pearson coefficient.

Now, we focus on the limiting case ($\gamma \gg 1$), for which the probability *p*_*k*_ to have nodes with degree *k K*_*min*_ is very low, see Eq. (2). Thus, we obtain networks formed only by nodes with degrees *K*_*min*_ and 1. Knowing that the total number of nodes is *N* and the total number of links is *N* − 1 one can show that the number of nodes with degree *K*_*min*_ equals ${N}_{{K}_{min}}=\frac{N-2}{{K}_{min}-1}$ and the number of nodes with degree 1 is ${N}_{1}=\frac{N({K}_{min}-\mathrm{2)}+2}{{K}_{min}-1}$. Using these results we are able to compute the sums from Eq. (12), which in this limiting case ($\gamma \gg 1$) are independent of *K*_*max*_ and take the following forms:

13 $$\begin{array}{l}{S}_{1}\,=\mathrm{2(}N-\mathrm{1)}\\ {S}_{2}\,=N({K}_{min}+\mathrm{2)}-\mathrm{2(}{K}_{min}+\mathrm{1)}\\ {S}_{3}\,=N({K}_{min}^{2}+{K}_{min}+\mathrm{2)}-\mathrm{2(}{K}_{min}^{2}+{K}_{min}+\mathrm{1)}\\ {S}_{e}\,=4N{K}_{min}-2{K}_{min}({K}_{min}+\mathrm{2).}\end{array}$$

Inserting Eq. (13) into Eq. (12) leads to the analytical expression of the Pearson coefficient:

14 $$C=-\frac{{[N({K}_{min}-\mathrm{2)}+2]}^{2}}{{K}_{min}[{N}^{2}({K}_{min}-\mathrm{2)}-2N({K}_{min}-\mathrm{3)}-4]}\mathrm{.}$$

For very large *N*, Eq. (14) reduces to:

15 $$C=-1+\frac{2}{{K}_{min}}\mathrm{.}$$

For *K*_*min*_ = 2, in the limiting case ($\gamma \gg 1$) one obtains a linear chain with *N* nodes, thus there are 2 nodes with degree 1 representing the chain ends, respectively *N* − 2 nodes with degree 2 representing the inner nodes of the chain. Its exact value of the Pearson coefficient, also obtained from Eq. (14), is *C* =  −1/(*N* − 2)≈ − 0.001 for *N* = 1000, in very good agreement with the results shown in Fig. 4. For *K*_*min*_ = 3 we obtain a sequence of connected nodes with degree *K*_*min*_ and peripheral nodes with degree 1. Its Pearson coefficient equals −0.334, comparable with the approximate value −1/3, calculated from Eq. (15).

### Eigenvalue spectra

**Figure 5:**
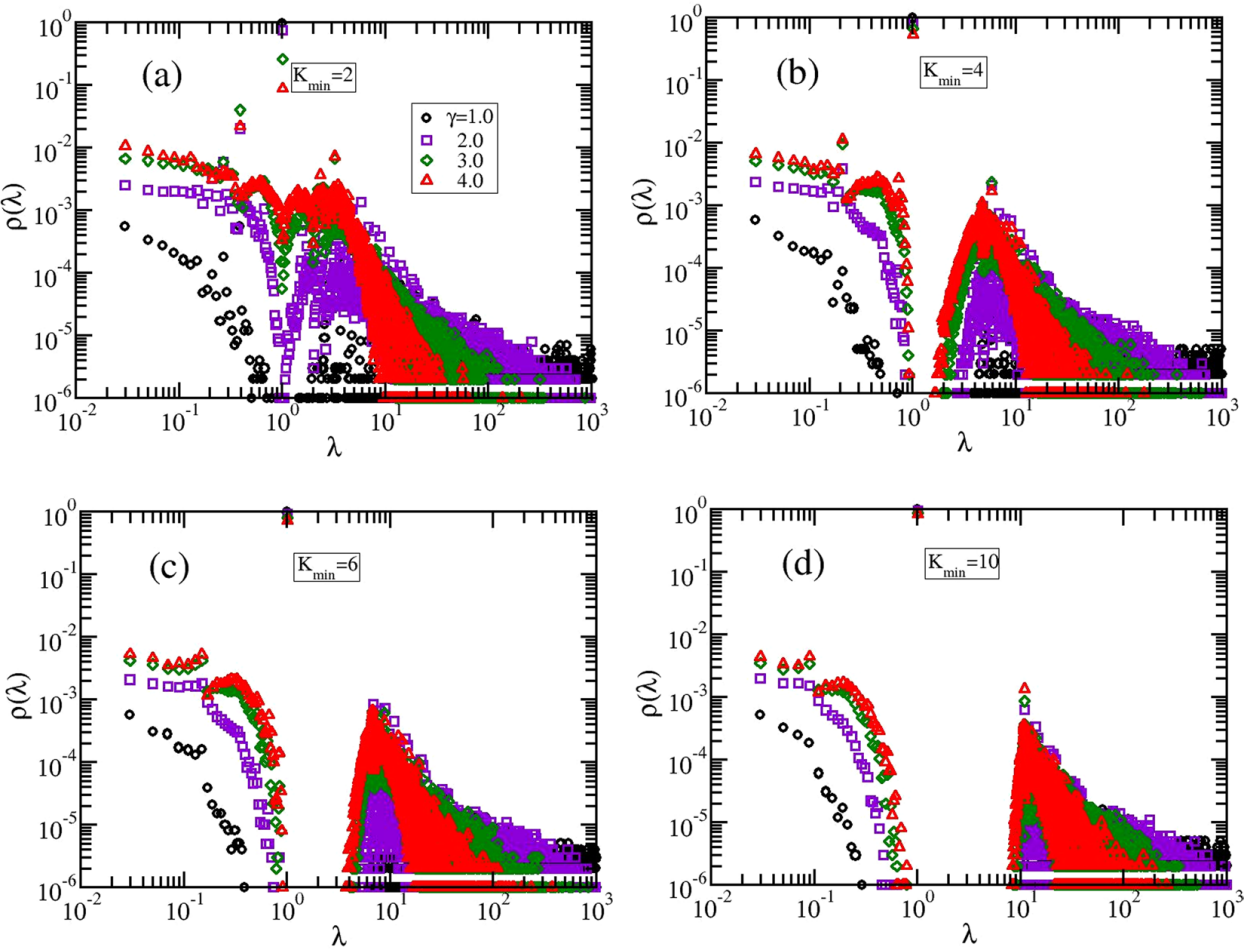
Eigenvalues density for GSFPNs with *N* = *K*_*max*_ + 1 = 1000 and different *K*_*min*_.

In Fig. 5 we focus on the influence of the minimum allowed degree *K*_*min*_ on the eigenvalue spectrum. Here we choose the maximum allowed degree *K*_*max*_ equal to *N* − 1 = 999 and *K*_*min*_ runs to 2, 4, 6, and 10. For all these values we display the results of *S* = 1000 realizations of GSFPNs with *γ* equal to 1, 2, 3, and 4. It is important to remind that *γ* = 1.0 provides networks with a more coupled starlike geometry, while for high values of *γ* we get networks formed mainly by nodes with degree *K*_*min*_. For all values of *K*_*min*_ we observe two distinct regions in the spectrum: a power-law behavior with exponent 0.5 for low eigenvalues (*λ* < 1), similar as the linear chain spectrum and a Poisson distribution decay in the region of higher eigenvalues (*λ* > 1). For all the values of *K*_*min*_ and *γ* one can easily notice a pronounced peak for *λ* = 1.0, which is due to an increase in the starlike segments. For instance, a star with *N* − 1 arms has three eigenvalues: *λ*_1_ = 0, *λ*_*N*_ = *N*, and the (*N* − 2)-fold degenerated eigenvalue *λ*_2,…, *N*_ − _1_ = 1. The number of appearances of eigenvalue *λ* = 1 diminishes by increasing the value of *γ*, which corresponds to more linear (*K*_*min*_ = 2) or dendritic-like (*K*_*min*_ ≥ 3) segments. This peak is followed by a gap between *λ* = 1 and the next higher eigenvalue, which becomes broader as the parameter *K*_*min*_ increases and *γ* is smaller. For example, for *K*_*min*_ = 10, Fig. 5(d), the above mentioned gap is equal to Δ*λ* ≈ 10. The presence and the length of this gap will have a direct influence on the relaxation quantities studied in Relaxation patterns. It is important to stress that the slopes of the relaxation quantities depend on the spectral dimension, *d*_*s*_, which can be related to the eigenvalues density through^64^

16 $$\rho ({\rm{\lambda }})\propto {{\rm{\lambda }}}^{{d}_{s}/2-1}\mathrm{.}$$

In the region of low eigenvalues and for GSFPNs with high *γ* the well-known result for linear chains are recovered, *ρ*(λ) ∝ λ ^−1/2^.

**Figure 6:**
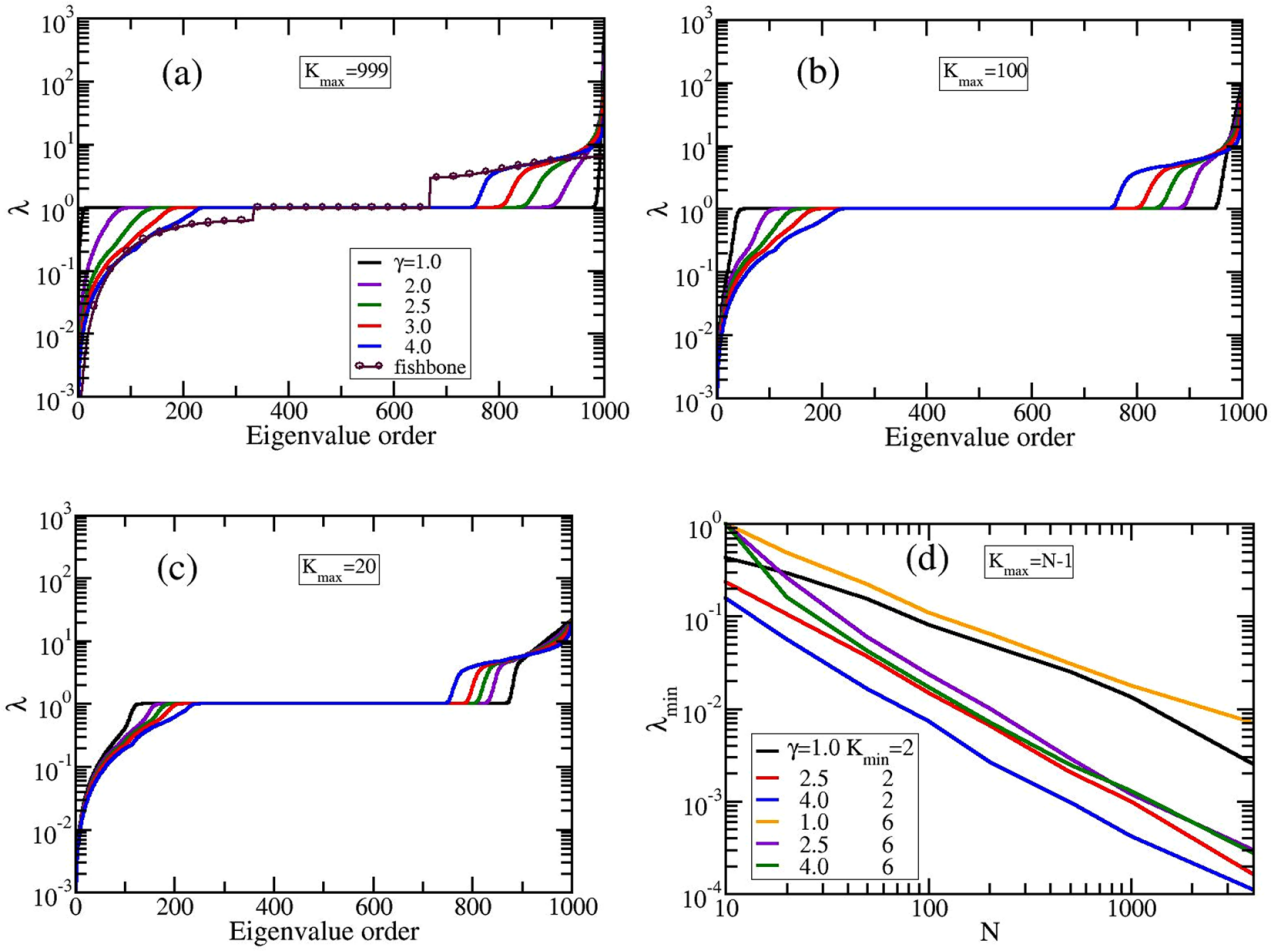
(**a**–**c**) Eigenvalues in progressive order for GSFPNs with *N* = 1000 and *K*_*min*_ = 4. (**d**) Lowest nonzero eigenvalue as a function of the network size.

In Fig. 6 (a–c) we plot the eigenvalues in progressive order for networks consisting of *N* = 1000 nodes and averaged over *S* = 1000 realizations. By keeping *K*_*min*_ constant to 4, we investigate the influence on the eigenvalue spectrum of the other parameter, *K*_*max*_. For this value of *K*_*min*_ the limit of very high *γ* corresponds to a network of nodes with functionality 4 linked together in a fish-bonelike or dendriticlike manner or a combination between these two. In this figure the parameter *K*_*max*_ is equal to *N* − 1, Fig. 6(a), 0.1 ⋅ *N* = 100, Fig. 6(b), and 0.02 ⋅ *N* = 20, Fig. 6(c). For a better illustration of the results we display in Fig. 6(a) also the spectrum of a fish-bone structure with functionality 4 of *N* = 1001 nodes. We notice that the influence of *K*_*max*_ is mainly relevant for GSFPNs with low *γ*. For larger values of *γ* the maximum allowed degree, *K*_*max*_, doesn’t play an important role, due to the fact that the networks are similar, namely they are structures built by nodes with the same degree, *K*_*min*_. By decreasing the value of *K*_*min*_ the degeneracy of the eigenvalue *λ* = 1 gets lower. For the same *K*_*max*_ the degeneracy of the eigenvalue *λ* = 1 diminishes when *γ* gets higher. The lowest nonvanishing eigenvalue equals *λ*_*min*_ ≈ 0.001, except for GSFPNs with *K*_*max*_ = 999 and *γ* = 1.0 or 2.0, Fig. 6(a), for which we have 0.039 and 0.002, respectively. The highest eigenvalue is more *K*_*max*_-dependent, for instance in the case of *γ* = 1.0 we have *λ*_*max*_ ≈ 584,93, and 21 for *K*_*max*_ = *N* − 1,100, and 20, respectively. In Fig. 6(d) we focus on how the lowest nonvanishing eigenvalue *λ*_*min*_ depends on the structure size, *N*. Here we display the results for *K*_*max*_ = *N* − 1 and *K*_*min*_ = 2 and 6 for three values of *γ*: 1.0, 2.5, and 4.0. We observe that *λ*_*min*_ scales for *γ* = 2.5 with the exponent equal to  − 1.23 (for *K*_*min*_ = 2) and  − 1.17 (for *K*_*min*_ = 6) and for GSFPNs with *γ* = 4.0 the exponent equals −1 for both *K*_*min*_-values. No clear scaling is observed for networks with *γ* = 1.0. The eigenvalue spectrum plays an important role in the relaxation patterns, Relaxation patterns.

### Relaxation patterns

**Figure 7:**
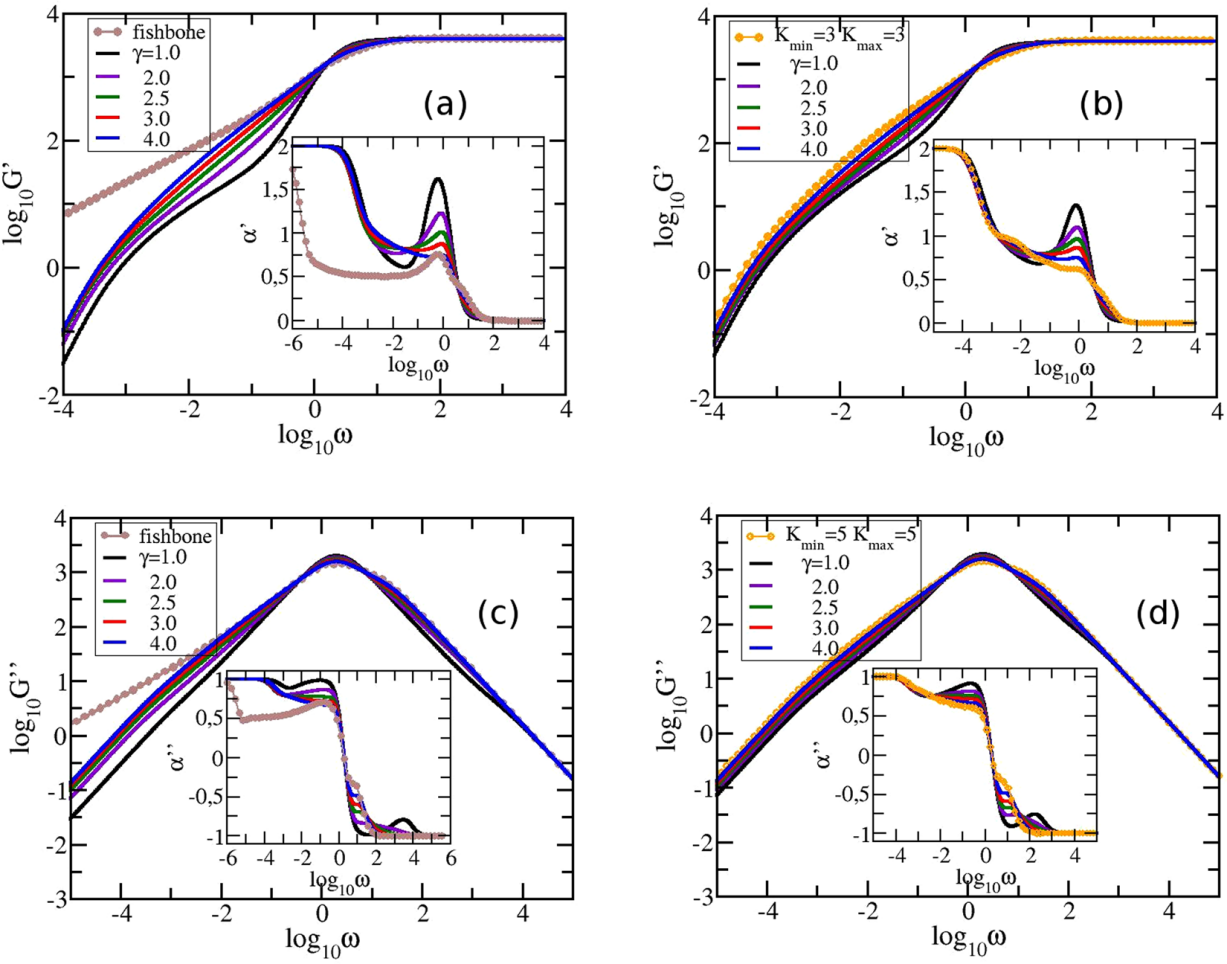
The storage modulus (upper row) and the loss modulus (lower row) for GSFPNs with *N* = 4000. The parameters’ set (*K*_*min*_, *K*_*max*_) is equal to: (**a**) (3, 400), (**b**) (3, 80), (**c**) (5, 3999), and (**d**) (5, 200).

Most measurements on polymers are monitored in the frequency domain; furthermore they involve macroscopic changes. Given the relative ease by which mechanical relaxation measurements can be nowadays performed, we focus on the moduli *G*′(*ω*) and *G*′′(*ω*), given by Eqs (10) and (11), where we set *νk*_*B*_*T*/*N* = 1 and *σ* = 1. Figure 7 shows the behavior of the storage modulus, *G*′(*ω*), and of the loss modulus, *G*′′(*ω*), calculated for GSFPNs of size *N* = 4000 and averaged over ensembles consisting of *S* = 250 realizations. The minimum allowed degree, *K*_*min*_, was kept constant to 3 for *G*′(*ω*) and to 5 for *G*′′(*ω*) and *γ* was varied: *γ* = 1.0, 2.0, 2.5, 3.0, and 4.0. The scales on all panels of the figure are double logarithmic to basis 10. We start our analysis with the storage modulus, panels 7 (a) and 7 (b). For comparison reasons we show in panel (a) the results achieved for a fish-bonelike structure, more precisely a structure with monomers of functionality 3 coupled to each other in a linear manner. These kind of structures are some special case of a larger class of networks: comb networks, which were experimentally synthesized^65^ or theoretically studied in polymer physics^66^ and reaction-diffusion problems^67,68^. Comb networks are composed of a linear chain, the *backbone*, and two side chains attached to every node of the backbone, the *teeth*. For example, if one considers the size of the side chains equal to 1 we obtain a fish-bonelike structure with functionality 4. This particular network can be obtained from our algorithm by choosing *K*_*min*_ = *K*_*max*_, but with an additional strong restriction: a maximum of two nodes with the same degree can be connected. For our considered fish-bone structure we obtain a clear region with slope 0.5, which is a trademark of linear chains. In panel (b) we display by symbols the results for the limiting situation *K*_*min*_ = *K*_*max*_ = 3, which is equivalent to structures formed by inner monomers with functionality 3 and peripheral monomers with functionality 1 organized in a random fashion. In Fig. 7(a) we restricted the maximum allowed degree to *K*_*max*_ = *N*/10 = 400 and in Fig. 7(b) *K*_*max*_ = *N*/50 = 80. Evidently in these two panels are the limiting, connectivity-independent behaviors at very small and very large frequencies; for $\omega \ll 1$ one has $G^{\prime} (\omega )\sim {\omega }^{2}$ which represents the mechanical response of the entire polymer network, whereas for $\omega \gg 1$ one finds $G^{\prime} (\omega ) \sim {\omega }^{0}$ which signifies single-bead mechanical response. However, neither the very low nor the very high frequency domains are typical for the GGS under investigation. Typical for the topological details of the structure under investigation is the intermediate frequency domain. The shape of the curves in the intermediate frequency domain suggests that different types of networks have been formed as function of parameter *γ*. For *γ* = 1 and 2 the intermediate frequency domain splits into two regions which suggests that the achieved networks consists of two major components. Moreover, the splitting of the intermediate domain highlights the existence of two relaxation processes, each component of the network relaxes on its frequency range independent of the other component. For values of *γ* larger than 2 the intermediate domain does not split which suggests that the obtained networks are single-component. For a better visualization of the intermediate frequency domain we show as inset the derivative $\alpha ^{\prime} =\frac{d({log}_{10}G^{\prime} )}{d({log}_{10}\omega )}$ as a function of ${log}_{10}\omega$. We observe that only by decreasing the maximum allowed degree, *K*_*max*_, the curves corresponding to different values of *γ* tend to stick to each other and the overall *γ*-dependent behavior is maintained. However, the curves get more distinct for smaller *γ*s than for higher *γ*s. For *γ* ≤ 2.5 we notice a peak at ${log}_{10}\omega \approx -0.21$, which starts to fade away when *γ* increases. Remarkably for *γ* = 3.0 we obtain a constant slope for almost two orders of magnitude with *α*′ ≈ 0.81 for *K*_*max*_ = 400 and *α*′ ≈ 0.79 for *K*_*max*_ = 80. This could mean that the achieved networks may be self-similar. Based on theoretical grounds, the spectral dimension^64^ of the self-similar networks is related to the power-law exponent (slope of the curve) through the relation *α*′ = *d*_*s*_/2. The estimated spectral dimension of the networks with *K*_*max*_ = 400 is *d*_*s*_ = 1.62 and for the networks with *K*_*max*_ = 80 is *d*_*s*_ = 1.60. We noticed that the width of this slope is maintained when *K*_*max*_ is varied. For *γ* ≥ 4.0 we get a more randomly branched structure, with maximum functionality *K*_*min*_ = 3, and the scaling disappears.

Now, we turn our attention to the loss modulus, *G*′′(*ω*). In panel 7 (c) we fixed the maximum allowed degree *K*_*max*_ to *N* − 1 and in panel 7 (d) *K*_*max*_ = *N*/20. In the same fashion as before, the left panel (c) also displays the loss modulus for a fish-bone structure of size *N* = 4002 and having the monomers with functionality 5. In panel (d) we also show by symbols the results for GSFPNs with *K*_*min*_ = *K*_*max*_ = 5 and *N* = 4002, which are structures with randomly connected monomers of functionality 5 (for inner monomers) and 1 (for peripheral monomers). Immediately apparent are the limiting behaviors of *G*′′(*ω*): a linear increase, *ω*^1^, for low frequencies and a *ω* − ^1^ dependence for high frequencies. Again, of our interest is the intermediate frequency domain where the topology of the structure reveals. Similarly to the case of storage modulus discussed above, we found also for *γ* = 1 and 2 that the in-between frequency domain of the loss modulus decomposes into two regions, showing clearly a two-components networks and the existence of two independent relaxation processes. For values of *γ* larger than 2 the in-between frequency domain does not decompose and one obtains single-component networks. In order to render the analysis more quantitative we present as inset the derivative, $\alpha ^{\prime \prime} =\frac{d({log}_{10}G^{\prime \prime} )}{d({log}_{10}\omega )}$, of the curves from the main figure. As we noticed for the storage modulus, when *K*_*max*_ is decreased the curves corresponding to different values of *γ* tend to a type of similar master curve, maintaining the same pattern. However, the curves that correspond to smaller values of *γ* are more different than the curves for higher *γ*s. Remarkably, in the case of *G*′′ we observe a better scaling for GSFPNs with *γ* = 2.5. This constant slope is similar for both panels and it is extended for almost two orders of magnitude and equals *α*′′ ≈ 0.77. From the value of the slope we determine the spectral dimension of these networks to be 1.54, a value closer to the one obtained from the analysis of the storage modulus. For *γ* ≥ 3.0 this constant slope region disappears. In the frequencies’ interval ${log}_{10}\omega \,\in \,\mathrm{(1,\; 3)}$ we observe a very prominent peak for *γ* = 1.0, which is shifted towards lower frequencies and in the same time is transformed into a small plateau when *γ* increases. This behavior was spotted before^8^ in the particular case of SFNs with *K*_*min*_ = 2 and *K*_*max*_ =  = *N* − 1 and it is due to the presence of more starlike segments in the network. The values of the slopes are similar with experimental values, namely 0.75, encountered for cross-linked polymer gel based on reversible covalent acylhydrazone bond^69^ and 0.8, found for Poly(vinyl chloride) plastisol gels^70^.

**Figure 8:**
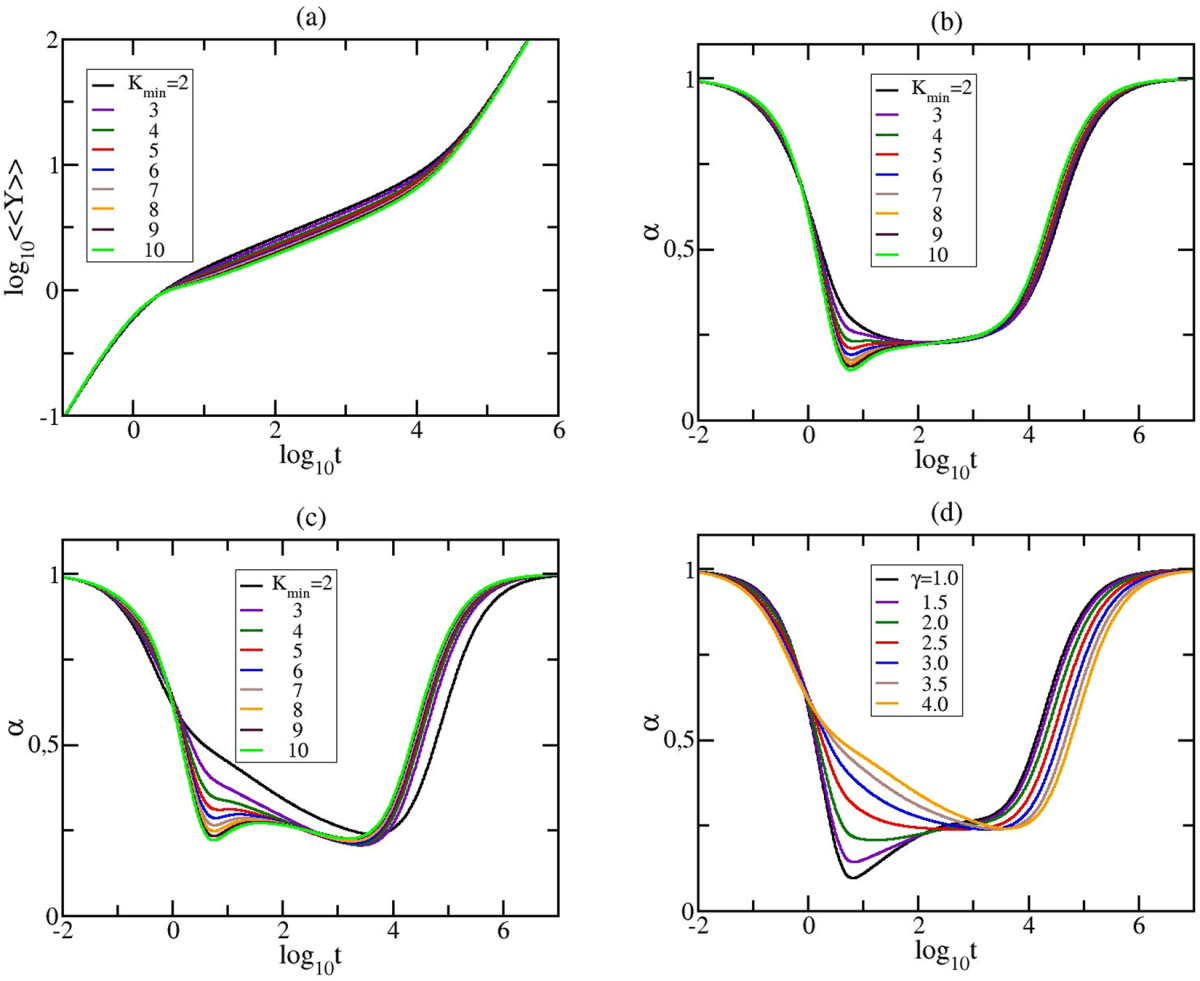
The average monomer displacement and its derivative for GSFPNs with *N* = 4000. The above row (**a**) and (**b**) corresponds to *γ* = 2.5, *K*_*max*_ = *N* − 1. In panel (**c**) the fixed parameters are: *γ* = 4.0 and *K*_*max*_ = 3999 and in panel (**d**) we have *K*_*max*_ = 200 and *K*_*min*_ = 2.

In Fig. 8 we plot in double logarithmical scale the average monomer displacement for GSFPNs with *N* = 4000. In the upper row of the figure we focus on the role of *K*_*min*_, keeping unchanged the other two parameters: *K*_*max*_ = 3999 and *γ* = 2.5. In Fig. 8(a) we present the results obtained for 〈〈*Y*〉〉 for the minimum allowed degree *K*_*min*_ ranging from 2 to 10, while in Fig. 8(b) we show their derivatives, $\alpha =\frac{d({log}_{10}\langle \langle Y\rangle \rangle )}{d({log}_{10}t)}$. Given that the scales of Fig. 8(a) are doubly-logarithmic, one sees that in the very short times domain one has $\langle \langle Y(t)\rangle \rangle \sim t$ which is due to the diffusive motion of single beads. On the other hand, at long times one reaches the domain $\langle \langle Y(t)\rangle \rangle \sim t/N$, which indicates that the structure moves as a whole and in the absence of an external field, based on the Einstein relation for GGS^14,26^ is the hallmark of simple diffusion. The most interesting situation corresponds to the intermediate time domain. For the chosen parameter set we obtain a constant slope region *α* ≈ 0.22 for *K*_*min*_ = 2 for times between ${log}_{10}t\mathrm{=1.5}$ and 3. Remarkably we observe that if the value of *K*_*min*_ increases the intermediate time region with this slope gets larger by almost one order of magnitude for *K*_*min*_ ≤ 5; the best scaling continues to be *α* ≈ 0.22 and it is obtained for *K*_*min*_ = 4. This finding can be related to the appearance of a gap in the eigenvalues’ density, feature shown in Fig. 5. For the average monomer displacement, the analytical expression that relates the spectral dimension with the power-law exponent is *α* = 1 − *d*_*s*_/2. Inserting the value of the slope in the beforehand power-law relation we determine the spectral dimension to be *d*_*s*_ = 1.56. This value is closely related to the spectral dimension of infinite combs embedded in 2*D* (*d* = 2):^67^ *d*_*s*_ = 2(1 − 2 ^*−d*^) = 3/2. The obtained value is in very good agreement to those determined from the analysis of the mechanical moduli. The achieved behavior for the average monomer displacement, together with the results obtained for the mechanical relaxation moduli harden the idea that for certain values of the parameter set one may obtain self-similar networks. For *K*_*min*_ > 5 the presence of more nodes with high degrees destroys the scaling in the intermediate time region and a local minimum emerges around ${log}_{10}t\approx 1$. The phenomenon of enlarging the width of an already existent scaling or the appearance of a new one was found also for GSFPNs that do not show scaling in the intermediate time region for *K*_*min*_ = 2. It is important to stress that this fact was encountered only for networks with *γ* ≥ 2.5. For GSFPNs with *γ* < 2.5 we observe no scaling for all *K*_*min*_-values. In Fig. 8(c) for a better visualization we display only the derivative *α* for GSFPNs with *γ* = 4.0. For this *γ*-value we do not have scaling for *K*_*min*_ = 2, but when the minimum allowed degree *K*_*min*_ is increased we encountered a new scaling region ranging for almost two orders of magnitude, equal to *α* ≈ 0.29 and obtained for *K*_*min*_ = 6. In Fig. 8(d) we focus on the influence of *γ*, keeping constant *K*_*max*_ to *N*/20 = 200 and *K*_*min*_ = 2. For a better visualization of the local slopes we show only the derivative *α*. We notice scaling of more than three orders of magnitude for GSFPNs with *γ* = 2.5, in agreement with our findings^8^ for the particular GSFPNs with *K*_*max*_ = *N* − 1. For *K*_*max*_ = 200 the value of *α* equals 0.24, while for *K*_*max*_ = 3999 was observed a scaling with *α* = 0.22. The value of the slopes obtained for the average monomer displacement is in good accordance with the value 0.25 of the anomalous diffusion exponent reported in^71^ for the Brownian motion of colloidal spheres in aqueous poly(ethylene oxide) solutions.

Remarkably, our theoretical findings are well supported by mechanical experiments performed on different types of polymers with topologies closer to our investigated networks. With respect to the division of the intermediate domain into two regions, similar behaviors with those obtained be us have been reported for POSS polymers^47^, complex supramolecular dendritic polymer networks in melt state^50^, star polyisoprene melts^51^, and diblock copolymer micelles^52^.

## Discussion

In this work we have introduced a new type of treelike scale-free network by considering two new modularity parameters for the usual power-law degree distribution. The first parameter restricts the minimum allowed degree, *K*_*min*_, while the second one controls the maximum allowed degree, *K*_*max*_. Thus, the scale-free polymer networks studied in^8^ becomes only a particular case of our construction procedure: it corresponds to *K*_*min*_ = 2 and *K*_*max*_ = *N* − 1. In the first part of the paper we have analyzed the structural properties of networks, by computing relevant quantities, such as the degree distribution and the diameter. These quantities are directly connected with the average shortest path and the degree correlations. When *K*_*min*_ is increased we have noticed a decrease in the diameter and for *γ* = 2.0 the diameter shows a logarithmical dependence on *N*, similar as the dendrimers. For larger values of *γ* the diameter shows a linear dependence with the size, *N*. We have shown that for all choices of the parameters’ set (*γ*, *K*_*min*_, *K*_*max*_) the networks are disassortatives, namely the nodes with high degree tend to connect to nodes with low degree.

We have performed our analysis in the framework of generalized Gaussian structures model by employing the Rouse-type approach. Of help here is that in the Rouse-regime, the main relaxation patterns depend only on the eigenvalues, but not on the eigenvectors of the connectivity matrix. The eigenvalue spectrum has shown a strong dependence on *K*_*min*_ for GSFPNs of any *γ*-value. Increasing the value of *K*_*min*_ we have observed the appearance of a gap in the spectrum, located between *λ* = 1 and the next higher eigenvalue. This gap was encountered for all *γ*s and it gets broader as long as *K*_*min*_ increases. The influence of *K*_*max*_ on the eigenvalue spectrum is less pronounced. The most important feature, which is more evident for lower *γ*s, is a decrease in the degeneracy of the eigenvalue *λ* = 1 when *K*_*max*_ gets lower. The dynamics of the networks has been analyzed through the investigation of the dynamical behaviors of the average monomer displacement and of the mechanical moduli. As observed for the static properties, the parameter *K*_*min*_ has also a stronger influence on the dynamical properties of the networks than the parameter *K*_*max*_. We have shown that if only the parameter *K*_*max*_ is decreased the moduli for all *γ*-values tend to the same curve and usually the value of the slope is maintained. When we varied the parameter *K*_*min*_ we have observed for intermediate frequencies various regions of constant slopes for different values of the parameters set (*γ*, *K*_*min*_). For GSFPNs of size *N* = 4000 we have obtained constant slopes of almost two orders of magnitude. These strengthen the fact that for certain values of the parameter set one may obtain self-similar networks, their spectral dimension laying in the interval (1.54,1.62). In Relaxation patterns we have highlighted the power-law behavior in the intermediate frequency region for (*K*_*min*_, *γ*) = (3,3.0) and (5, 2.5). It is important to mention that similar scaling behavior can be observed for other values of *K*_*min*_ for an appropriate *γ*-value. In the analysis of the dynamical behavior of the average monomer displacement we have mainly concentrated on the influence of *K*_*min*_ for a particular choice of *γ*, specifically *γ* = 2.5. Varying the parameter *K*_*min*_, while *K*_*max*_ is fixed we have been able to increase the width of the scaling region by one order of magnitude, obtaining a larger power-law behavior for *K*_*min*_ = 4. This remarkable finding was extended to GSFPNs with higher *γ*s that doesn’t show scaling for *K*_*min*_ = 2, but will scale when *K*_*min*_ is increased to a certain value. As example, we have chosen to display this behavior for GSFPNs with *γ* = 4.0, which scales if *K*_*min*_ = 6. Remarkably, our theoretical findings are well supported by mechanical experiments performed on different types of polymers. We expect our findings to be important not only to polymer physics or related areas of research, but also to the research of complex real networks^56^, of classical and quantum transport on complex networks^72–75^, of the coherent transfer of excitons^76–78^ or of the fluorescence depolarization under quasiresonant Förster energy transfer^35,79^.
